# An analysis of the domestic resumption of social production and life under the COVID-19 epidemic

**DOI:** 10.1371/journal.pone.0236387

**Published:** 2020-07-22

**Authors:** Xinliang Xu, Shihao Wang, Jinhui Dong, Zhicheng Shen, Shuwan Xu

**Affiliations:** 1 State Key Laboratory of Resources and Environmental Information Systems, Chinese Academy of Sciences, Institute of Geographic Sciences and Natural Resources Research, Beijing, China; 2 University of Chinese Academy of Sciences, Beijing, China; 3 Tongzhou Campus, The High School Affiliated with Renmin University of China, Beijing, China; Institute for Advanced Sustainability Studies, GERMANY

## Abstract

Population migration and urban traffic are two important aspects of the socioeconomic system. We analyze the trends of social production and resumption of life after the coronavirus disease 2019 (COVID-19)-influenced Spring Festival in 2020 with statistics on reported cases of COVID-19 from China’s National Health Commission and big data from Baidu Migration (a platform collecting population migration data). We find that (1) the distribution of COVID-19 cases throughout mainland China has a specific spatial pattern. Provinces in eastern China have more reported cases than those in western China, and provinces adjacent to Hubei have more confirmed COVID-19 cases than nonadjacent provinces. Densely populated regions with well-developed economies and transportation are more likely to have cluster infection incidents. (2) The COVID-19 epidemic severely impacts the return of the migrant population in the Spring Festival travel rush, as demonstrated by the significant reduction in the return scale, along with the extended timespan and uncertainty regarding the end of the travel rush. Among 33 provinces, special administrative regions, autonomous regions and municipalities, 23 of them (approximately 70%) have a return rate below 60%. Hubei, Hong Kong, Xinjiang, and Inner Mongolia have the lowest return rates (below 5%), whereas the return rates in Hainan and Shandong, 272.72% and 97.35%, respectively, indicate the best trend of resumption. Due to government regulations, the population return in densely populated and well-developed regions shows a positive trend. (3) The resumption of urban traffic is slow and varies greatly in different regions. The urban traffic conditions in 22 provinces and municipalities have a more than 60% level of resumption. Guizhou and Yunnan have the highest level of resumption of urban traffic, whereas Xinjiang, Hubei, and Heilongjiang have the lowest (29.37%, 35.76%, and 37.90%, respectively). However, provinces and municipalities with well-developed intercity traffic have a lower level of resumption, mainly because of regulatory methods such as lockdowns and traffic restrictions. The increased public awareness of epidemic prevention and the decreased frequency of outdoor activities are also two positive factors slowing the spread of the epidemic. (4) Time will be necessary to fully resume social production and life throughout China. Xining and Jinan have the highest levels of resumption, 82.14% and 71.51%, respectively. Urumqi and Wuhan are the cities with the lowest levels of resumption, only 0.11% and 0.61%, respectively. Currently, 12 of 33 provinces and municipalities have levels of resumption of more than 80%; among them, Guizhou, Yunnan, and Gansu have with the highest levels of resumption and have nearly resumed the 2019 levels of work and life, whereas Xinjiang and Hubei have the lowest resumption rates, only 0.09% and 7.57%, respectively. Thus, relevant government departments should focus more on densely populated and well-developed provinces and cities when applying epidemic prevention and work resumption methods. We reveal the general conditions of the epidemic and the population return scale across China, along with urban traffic conditions and the resumption of social production and life under COVID-19, providing a scientific basis for local governments to make further decisions on work resumption.

## Introduction

At the end of 2019, an outbreak of coronavirus disease 2019 (COVID-19) occurred in Wuhan and spread nationwide. The high contagiousness of the disease has posed a serious challenge to people’s health of people and to their daily life and overall socioeconomic conditions. The government has taken multiple methods, including city-wide lockdowns, the suspension of public transportation, the monitoring of unplanned population migration, domestic self-isolation and extension of the Spring Festival holiday to firmly prevent the possible transmission of the virus [1]. As a large-scale epidemic, the COVID-19 outbreak has severely impacted various parts of society and caused many difficulties throughout the country. Economic activities in China have even been at a standstill for a short period of time. During the Spring Festival, the epidemic influenced major domestic consumption and service industries such as accommodations, retail, catering, entertainment, transportation and tourism. Delayed resumption of work has also had a huge impact on industry, manufacturing production and other sectors [2]. With many effective prevention and control measures, progress has been made in the fight against the COVID-19 epidemic. Since late February, employees in various industries have resumed their work, and social production and life in China are gradually resuming. In February 23, a conference on the overall promotion of prevention and control of COVID-19 and work toward economic and social development was held in Beijing. President Xi Jinping attended the conference and delivered an important speech. In March 4, the General Office of the State Council issued a notice focusing on further simplifying the process of production approval and optimizing governmental services to accurately and steadily promote the resumption of production. On March 12, the *Notice of Conducting an Investigation into the Resumption of Production* was issued. Prevention and control of the epidemic are currently emerging, and socioeconomic development remains our long-term goal. Only when we take environmental, economic and social factors into account can we win the battle against the epidemic. Currently, we have managed to control the epidemic, and a discussion of the resumption of production must follow. However, various difficulties and risks may exist in this process, requiring a longer period of time for complete resumption. What is the current process and status of the resumption of social production and life across the country? How can precise policy guidance be made for the problems existing in the next step? These questions are general concerns of governments at all levels.

Population migration refers to the process of people leaving their places of residence and going elsewhere for the purposes of working and living [3]. It is the process of the spatial reallocation of production factors [4] and is an important representation and carrier of regional social and economic activities. Population migration reflects the functional association between regions to some extent [5]. As a sure outcome of urban development, population migration also affects the process of urban economic and social development [6]. It narrows regional economic differences by both inspiring the social and economic development of the outflow area and promoting that of the inflow area [7]. Scholars in various fields have consistently focused on interregional population migration, a specific population phenomenon. Population migration affects not only the demography and society but also the economic development of a region. A region's level of economic development has a strong positive correlation with population mobility [8]. China is a country with significantly different regional income levels and a large population; thus, it is particularly important to explore the internal relationship between population migration and regional economic growth [9]. For example, Ding et al. constructed a dynamic panel data model in which population migration affects regional economic growth. Their empirical analysis found that an increase in net population inflow will significantly promote economic growth [9]. Tan et al. analyzed the effect of population migration on China's regional economic growth and found that interprovincial migration has different effects on promoting economic growth in different regions [10]. Chen et al. analyzed the main characteristics of China’s mobile population and its influence on China’s urbanization and found that the proportion of the mobile population is closely related to the level of regional urbanization. Population migration and movement have become the most important driving factors of China’s urbanization process [11].

At present, with the advancement and wide application of location-based service (LBS) technology supported by global systems for mobile communications (GSMs) and global positioning systems (GPSs), large-scale individual spatiotemporal data are more accessible to researchers [12] and are becoming an increasingly important medium for characterizing the geographic behavior of residents. This paper uses statistics on confirmed COVID-19 cases from the National Health Commission of the People’s Republic of China (PRC) and big data from Baidu Migration to analyze the distribution pattern of COVID-19 cases, the return of the population in various provinces and cities across the country and the spatiotemporal evolution of urban traffic, along with the resumption of social production and life after the 2020 Spring Festival, with a view to providing a reference and decision-making basis for comprehensive social resumption under the COVID-19 epidemic.

## Data and methods

### Data sources

Population migration and movement are activities in which production factors are reconfigured, and they can promote the reaggregation and diffusion of social and economic factors to a certain extent [13]. As the most essential factor in social production activities, population movement can reveal the flow of advanced production factors such as material, capital, information and technology as well as the trend of social and economic development [14]. During the Spring Festival travel rush, the country sees large-scale population movements, and multiple modes of transportation, such as airplanes, trains, and vehicles, make it more difficult to obtain precise statistics on the number of passengers and their destinations. With the advent of various LBS providers, big data based on individual travel records can function as valuable group behavior data for researchers in geography. These location-based data include the migration of non-household-registered populations and tourism movements, and they are time sensitive and continuous. With these advantages, they become fundamental for accurately analyzing the activities, social production and life conditions under the epidemic [15].

In this research, population mobility and traffic intensity data are collected from the Baidu Map Smart Eye—Baidu Migration Big Data platform (referred to as Baidu Migration). Baidu Migration is a big data platform launched by Baidu in 2014. It serves to reflect the paths and characteristics of population movement during the Spring Festival. Supported by frequent population migration and the high availability of mobile communication devices, it integrates big data, cloud computing and LBSs to track travel paths through mobile devices. The platform displays the daily population flow in real time and records the migration paths of hundreds of millions of people. Thus, we collect data from February 3, 2020, to March 6, 2020, from the platform. The time span starts immediately after the end of the Spring Festival holiday and includes four whole weeks and five weekdays of the fifth week (33 days in total). We also collect data related to the daily migration scale index and urban traffic intensity among provinces, municipalities and 360 cities across the country during the same period in the last year (from February 11, 2019, to March 15, 2019). Subsequently, an analysis of the resumption of social production and life under the COVID-19 epidemic is conducted based on a comparative analysis of these data. Data from the Baidu Migration Big Data Platform have been widely used in research in fields such as urbanization and population migration [5–6]. In addition, the socioeconomic data used in this paper are collected from the statistical yearbooks of relevant provinces.

### Methods

To analyze the population activities, social production and living conditions under the COVID-19 epidemic, this research mainly calculates the daily population migration scale index (*PMSI*) and urban traffic intensity index (*UT*) in the 5-week period after the end of the 2020 Spring Festival, comparing them with the data on the same period in 2019. *PMSI* reflects the size of the in- or outflowing population and can be compared synchronically between cities. *UT* refers to the indexed ratio of the population taking part in urban traffic to a city’s total residential population. Our purpose is to describe the resumption of social production and life under the influence of the epidemic via three main indicators: the population return scale index (*PRSI*), resumption of the scale of population return (*PRSI*_*re*_), and resumption of urban traffic (*UT*_*re*_). *PRSI* is defined as the difference between a city’s immigration and emigration indexes, and it reflects the size of a city’s returning population. A positive *PRSI* represents population inflow, whereas a negative value represents population outflow. *PRSI*_*re*_ and *UT*_*re*_ indicate the resumption of population return and urban traffic in 2020 compared to the previous year. The calculation of each index is as follows.

Population return scale index:

$$PRSI=\sum_{11}^{55}{PMSI}_{in}-\sum_{11}^{55}{PMSI}_{out}$$

where *PRSI* is the population return scale index and $\sum_{11}^{55}{PMSI}_{in}$ and $\sum_{11}^{55}{PMSI}_{out}$ are the sum of the population immigration scale index and the population emigration scale index from the Monday of the first week (February 3) to the Friday of the fifth week (March 6) after the Spring Festival holiday.

Resumption of the scale of population return:

$${PRSI}_{re}=\frac{{PRSI}_{2020}}{{PRSI}_{2019}}\times 100\%$$

where *PRSI*_*re*_ is the resumption of the scale of population return and *PRSI*_2020_ and *PRSI*_2019_ are the population return scale indexes for 2020 and 2019, respectively.

Resumption of urban traffic:

$${UT}_{re}=\frac{{\bar{UT}}_{2020}}{{\bar{UT}}_{2019}}\times 100\%$$

where *UT*_*re*_ is the resumption of urban traffic and ${\bar{UT}}_{2020}$ and ${\bar{UT}}_{2019}$ are the average urban traffic intensity from the Monday of the first week to the Friday of the fifth week after the end of the Spring Festival holiday in 2020 and 2019, respectively.

Dynamic changes in social production and life can be seen through population migration and urban traffic conditions. In this research, the resumption of social production and life order (*SPLO*_*re*_) is calculated based on the scale of population return and the intensity of urban traffic (both standardized) through a weighted sum. The resumption of the scale of population return and the resumption of urban traffic are two important aspects indicating a city’s work resumption, and there is no strong correlation (R^2^ = 0.1, P = 0.115) between them. Thus, we give the two indicators the same weight (0.5) for a simpler assessment of resumption. The equation is as follows:

$${SPLO}_{re}=\frac{{0.5{\times PRSI}_{2020}+0.5\times UT}_{2020}}{{0.5{\times PRSI}_{2019}+0.5\times UT}_{2019}}\times 100\%$$

where *SPLO*_*re*_ is the resumption of social production and life order.

## Results and analyses

### Statistics on total cases and their distribution in various provinces

**Fig 1:**
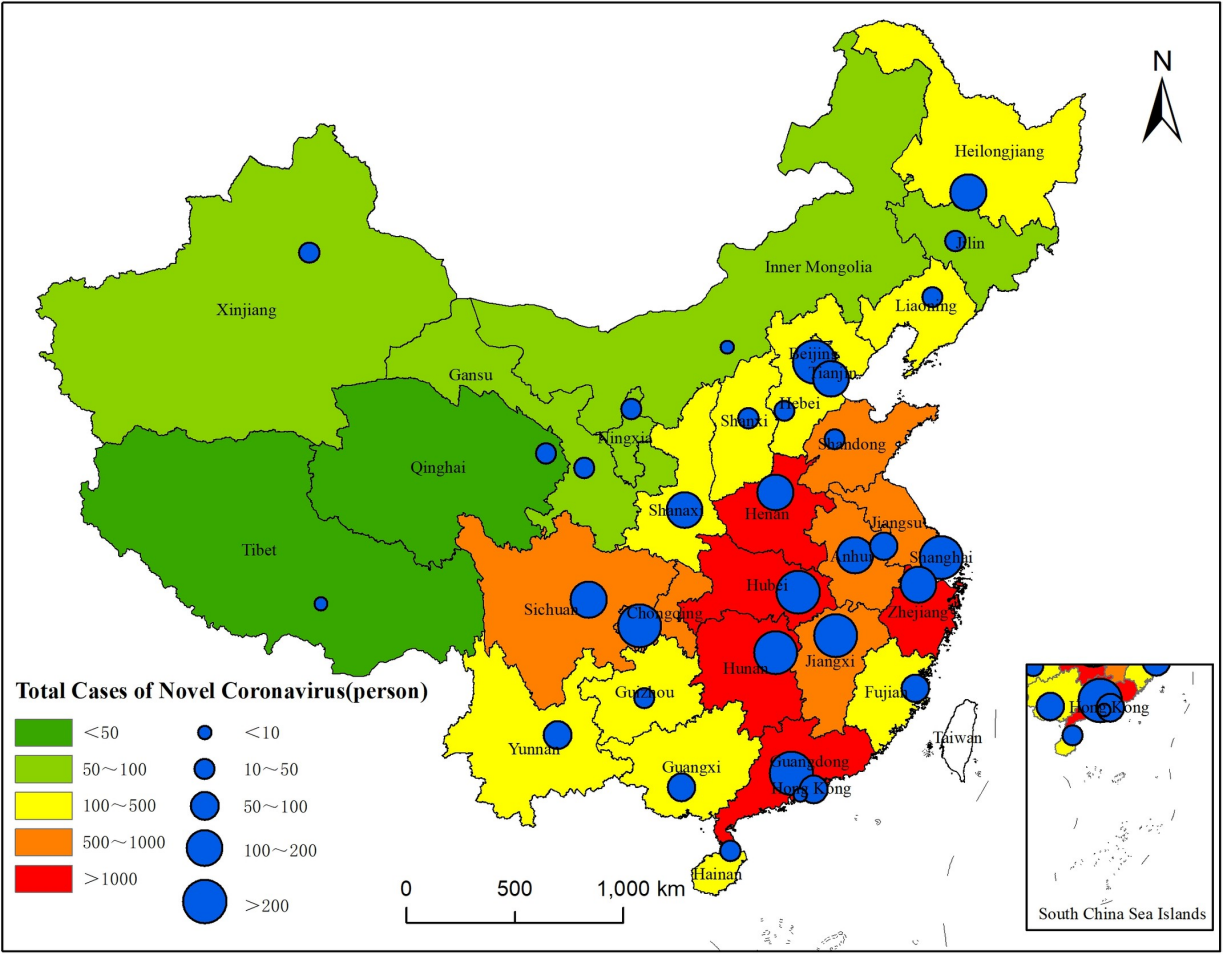
Distribution of the total number of confirmed cases of COVID-19 in provinces, municipalities and capital cities (statistics as of March 6, 2020).

By March 6, 2020, there were 80,651 confirmed cases of COVID-19 in China, including 67,666 cases in Hubei (with 49,871 of them being in Wuhan). Among 33 provinces and municipalities, 33% of them (11 of 33) reported more than 500 cases. The number of cases in Hubei, Hunan, Zhejiang, Guangdong and Henan exceeded 1000, thus making them the provinces with the largest number of total cases. In 24% (8 of 33) of provinces and municipalities, the number of cases was less than 100, and in Tibet and Qinghai, the number was below 20. Similarly, among the 33 provincial capital cities, Wuhan, Chongqing, Beijing, Guangzhou, Shanghai, Changsha, and Nanchang all reported more than 200 cases, while the number of cases in Lhasa, Hohhot, and Xining was less than 50. The spatial distribution of confirmed cases follows a special pattern: the cases are mainly found in Hubei and surrounding provinces, and there are more cases in eastern and southern China than in western and northern China (Fig 1).

### The resumption of population return

**Fig 2:**
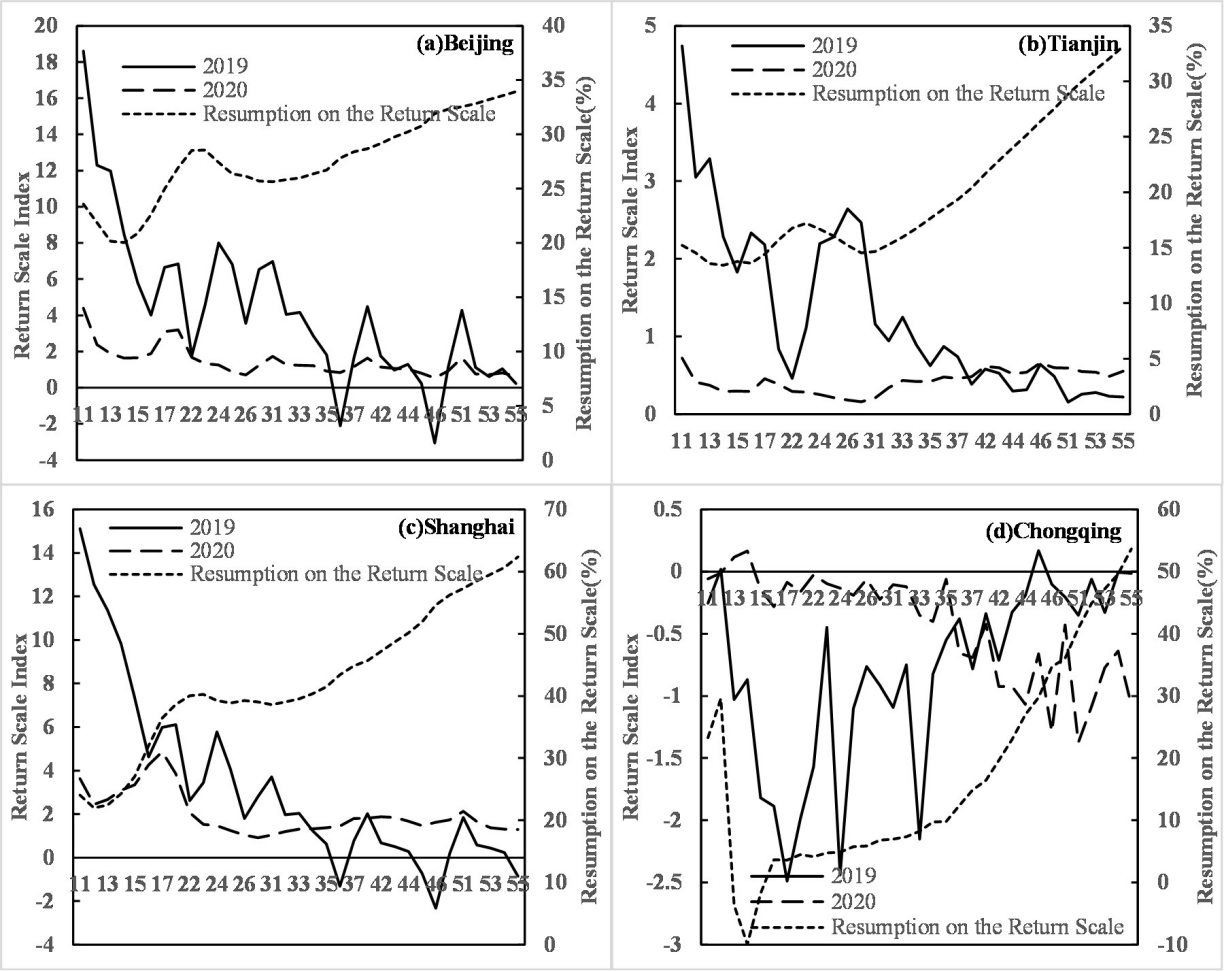
Daily changes in the return scale index and resumption of return scale in Beijing, Tianjin, Shanghai and Chongqing.

The COVID-19 epidemic in 2020 severely impacted the latter part of the Spring Festival travel rush (i.e., migrating people returning to their present places of residence), as demonstrated by its significant reduction in scale, along with the extension of the timespan and the uncertainty regarding the end of population migration. The population return charts for Beijing, Tianjin, Shanghai, and Chongqing (Fig 2) show that the resumption of population return after the Spring Festival holiday in Beijing and Tianjin is approximately 30%, reaching 33.17% and 33.95%, respectively. For Shanghai and Chongqing, the population return scale is larger, 62.39% and 53.65%, respectively, ranking highly compared to other provincial capital cities. The time process (Fig 2) shows that the population return in 2019 was generally completed within two weeks of the end of the Spring Festival holiday, while in 2020, the number of returning people remained low and stable, and there was no population return peak as there was in the previous year due to the COVID-19 epidemic. Measures such as traffic restrictions and other relevant policies and the delay in the resumption of business and school resulted in a greatly reduced population return scale compared to the 2019 level. The resumption of return scale generally shows a steady upward trend after the third week. From the population movement perspective, Chongqing is a city with a large population emigrant scale. Its population return scale index in 2019 was -26.56, but the index in 2020 was only -14.25, and the time when people returned to their present places of residence was also significantly delayed. In 2020, the fourth and fifth weeks after the Spring Festival holiday saw more emigrants in Chongqing compared to the previous three weeks. The statistics for the other 29 capital cities show that the population return scale for Chengdu, Hangzhou, Lhasa and Guangzhou had larger levels of resumption, 62.94%, 55.05%, 52.98% and 50.54%, respectively. Hong Kong, Wuhan, Urumqi, Shijiazhuang and Harbin had the lowest levels of resumption on the scale of population return (all below 10%), with Hong Kong, Wuhan, and Urumqi having the smallest levels of resumption, 2.78%, 3.88% and 4.10%, respectively.

**Fig 3:**
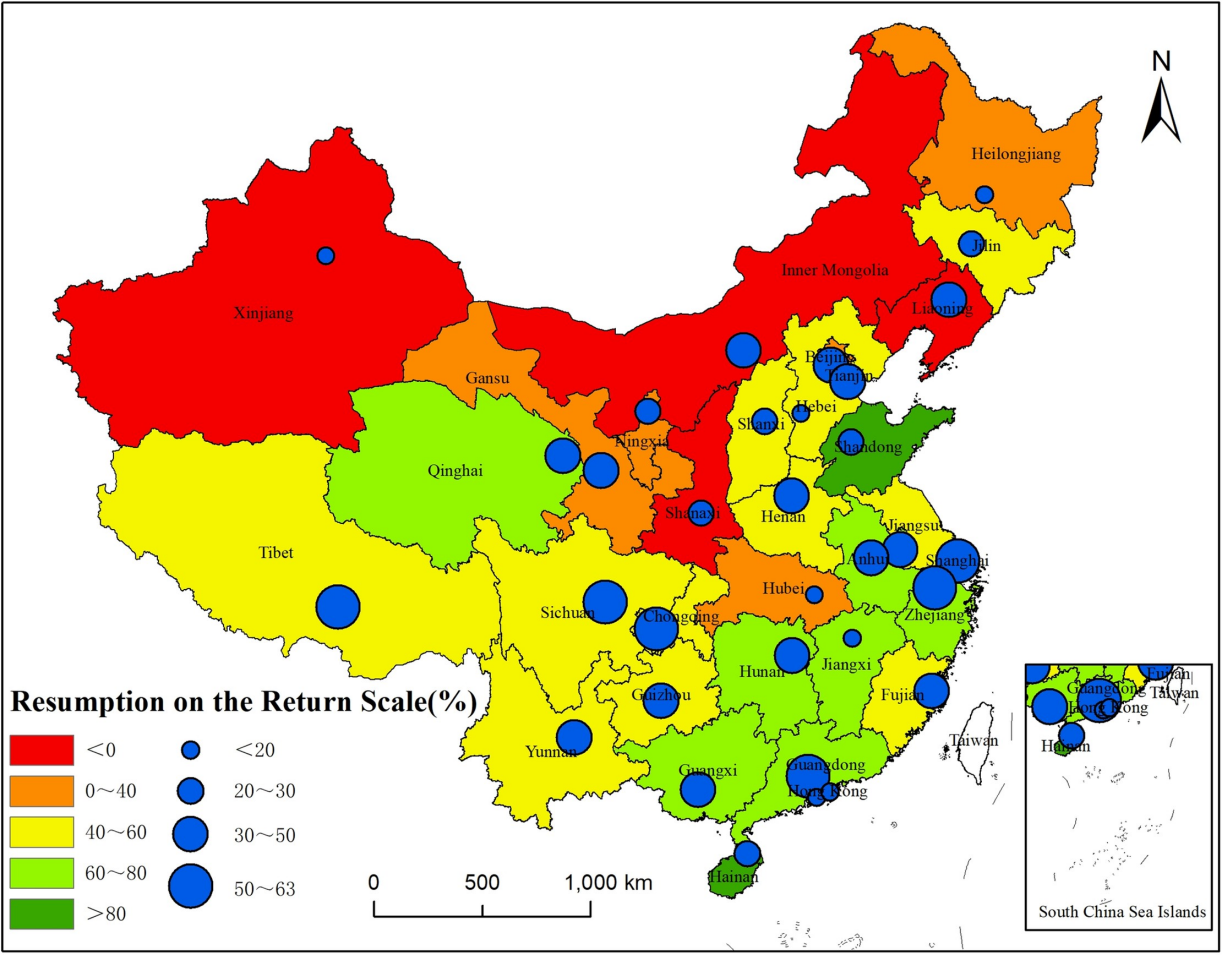
Distribution of the resumption of the scale of population return in provinces, municipalities and capital cities.

Regarding the overall population return of provinces and municipalities, the population return scale in 70% of them (23 of 33) is below 60%, and in 39% of them (13 of 33), it is below 50%. The COVID-19 epidemic impacted Hubei Province most severely, causing it to have the lowest level on the scale of population return (0.78%). The resumption levels in Hong Kong, Xinjiang and Inner Mongolia are all below 5%, 2.78%, -2.86%, and -4.35%, respectively. Macao, Shaanxi and Ningxia also have a scale of resumption less than 20%. From the population movement perspective, the population flows of Xinjiang, Inner Mongolia, Shaanxi and Liaoning in 2020 are the opposite of those in 2019, which can explain why the figures related to the resumption of the scale of population return in Fig 3 are negative. For example, in 2019, the population flow in Inner Mongolia mainly consisted of emigration, and the population return scale index was -8.58. However, after the Spring Festival holiday in 2020, Inner Mongolia showed a state of population immigration, and the index was 0.37. In addition, the resumption of the scale of population return in Hainan and Shandong Provinces is the most ideal among the 33 provinces and municipalities countrywide. The population return scale in Hainan reaches 272.72%, far exceeding the that for same period in 2019. The same figure in Shandong Province, 97.35%, is very close to the level in 2019. Qinghai and Hunan also see a scale of population return that is better than that of other provinces, 76.97% and 76.27%, respectively.

### The resumption of urban traffic

**Fig 4:**
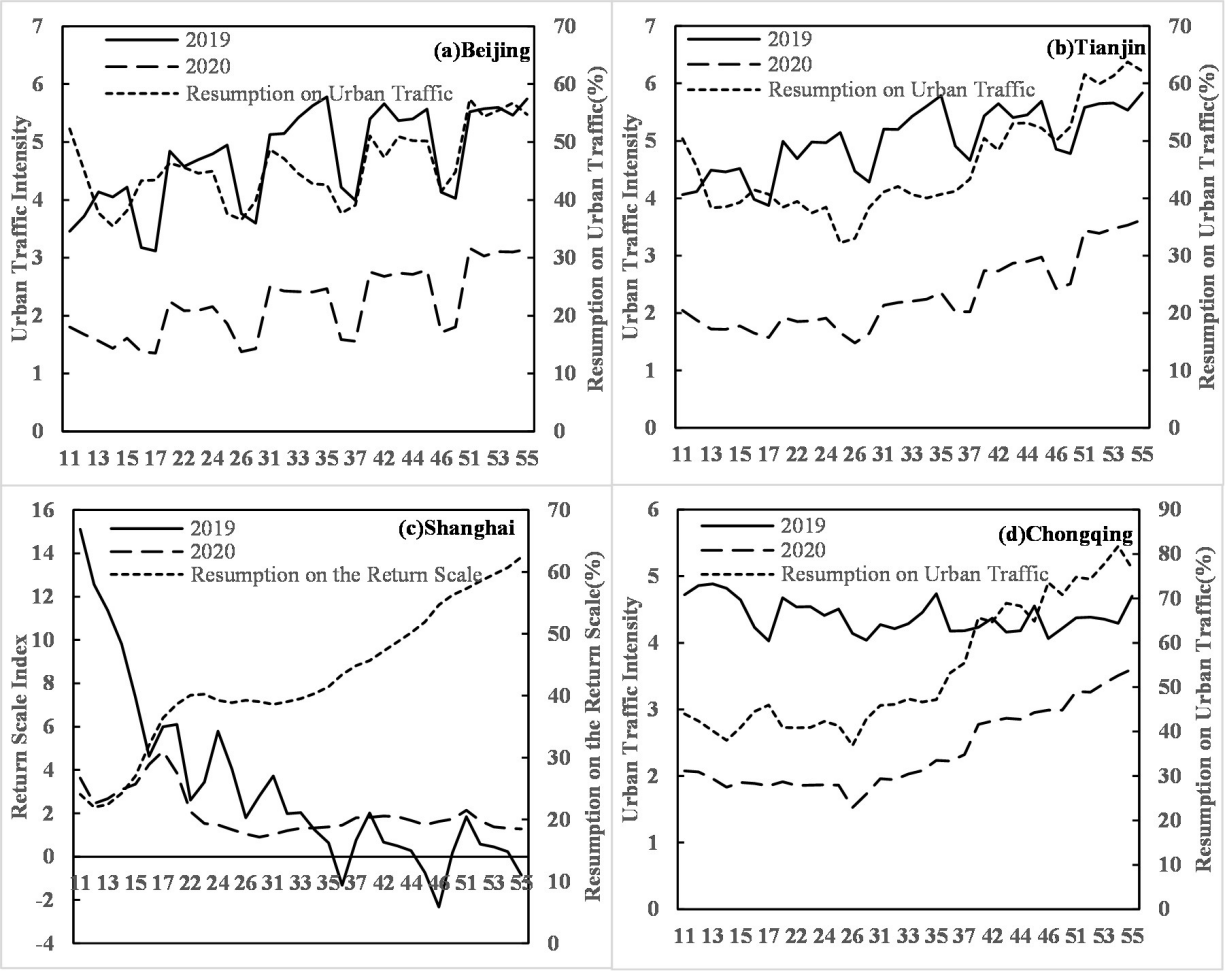
Daily changes in urban traffic intensity and the resumption of urban traffic in Beijing, Tianjin, Shanghai and Chongqing.

The resumption of urban traffic is slow and varies greatly in different places due to the epidemic. The resumption levels of urban traffic in Beijing and Tianjin are 46.31% and 46.23%, respectively, and the highest levels of daily resumption reach 57.37% and 63.78%, respectively. The same levels in Shanghai and Chongqing are slightly higher, 54.52% and 53.89%, respectively. Additionally, the highest levels of daily resumption in the two municipalities reach 77.84% and 81.73%, respectively (Fig 4). Among the other 29 provincial capital cities and special administrative regions, the resumption levels of 16 cities are more than 50%. Among them, Jinan, Xining and Hong Kong have the highest levels of resumption, exceeding 60% (at 63.83%, 62.45% and 61.86%, respectively), whereas Urumqi and Wuhan have the lowest levels of resumption, 14.25% and 15.19%, respectively.

**Fig 5:**
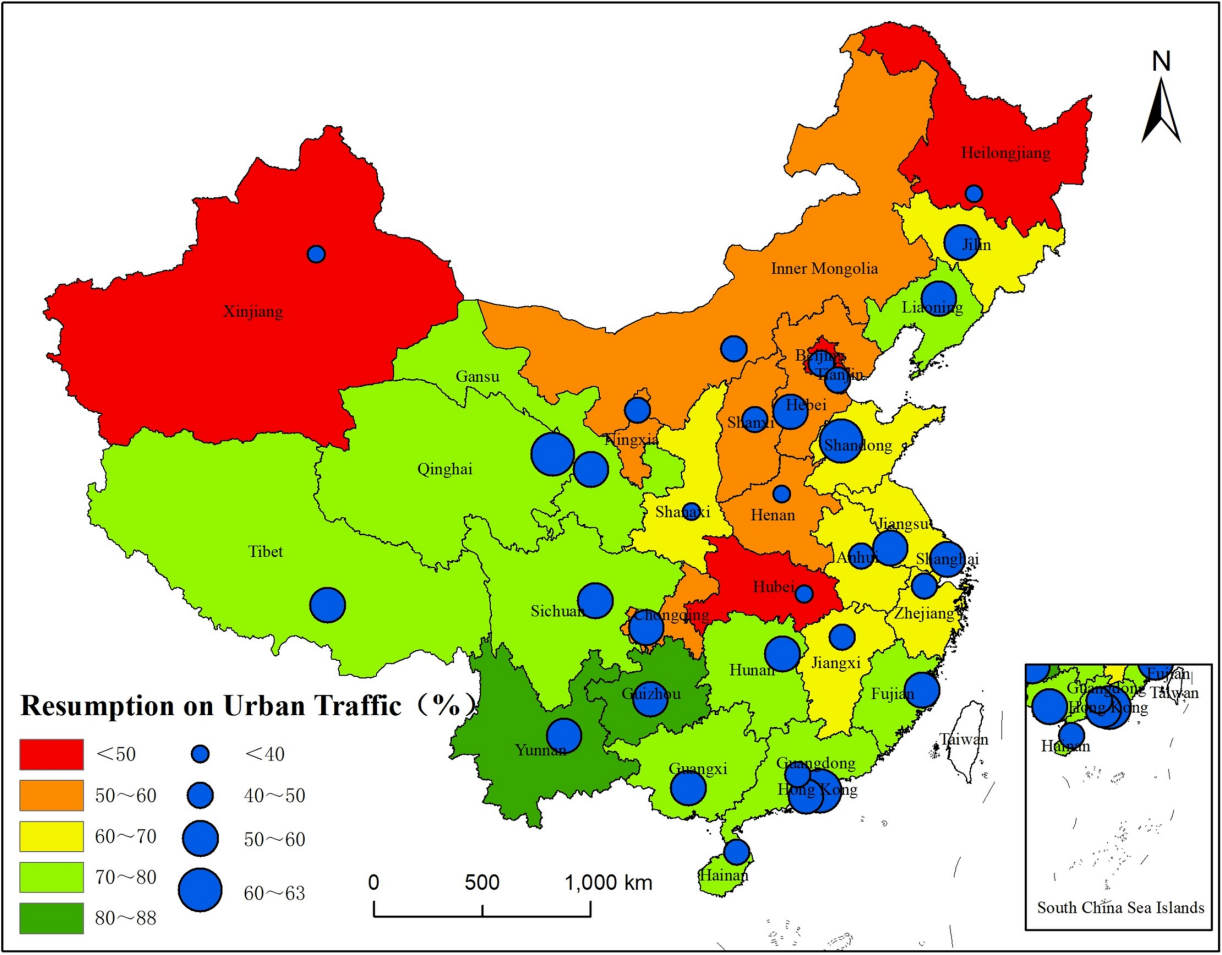
Distribution of the resumption of urban traffic in provinces, municipalities and capital cities.

The resumption of overall transportation in various provinces and cities shows (Fig 5) that 22 of 33 provinces and cities have resumption levels of more than 60%. The highest resumption levels can be found in Guizhou and Yunnan, with values of 87.76% and 83.22%, respectively. In addition, the overall transportation in 10 provinces and autonomous regions (Guangdong, Fujian, Qinghai, Liaoning, Guangxi, Gansu, Tibet, Hunan, Sichuan, and Hainan) has resumed to a level of more than 70%. The overall transportation resumption levels in Xinjiang, Hubei, and Heilongjiang are the lowest, only 29.37%, 35.76%, and 37.90%, respectively.

### The resumption of social production and life

**Fig 6:**
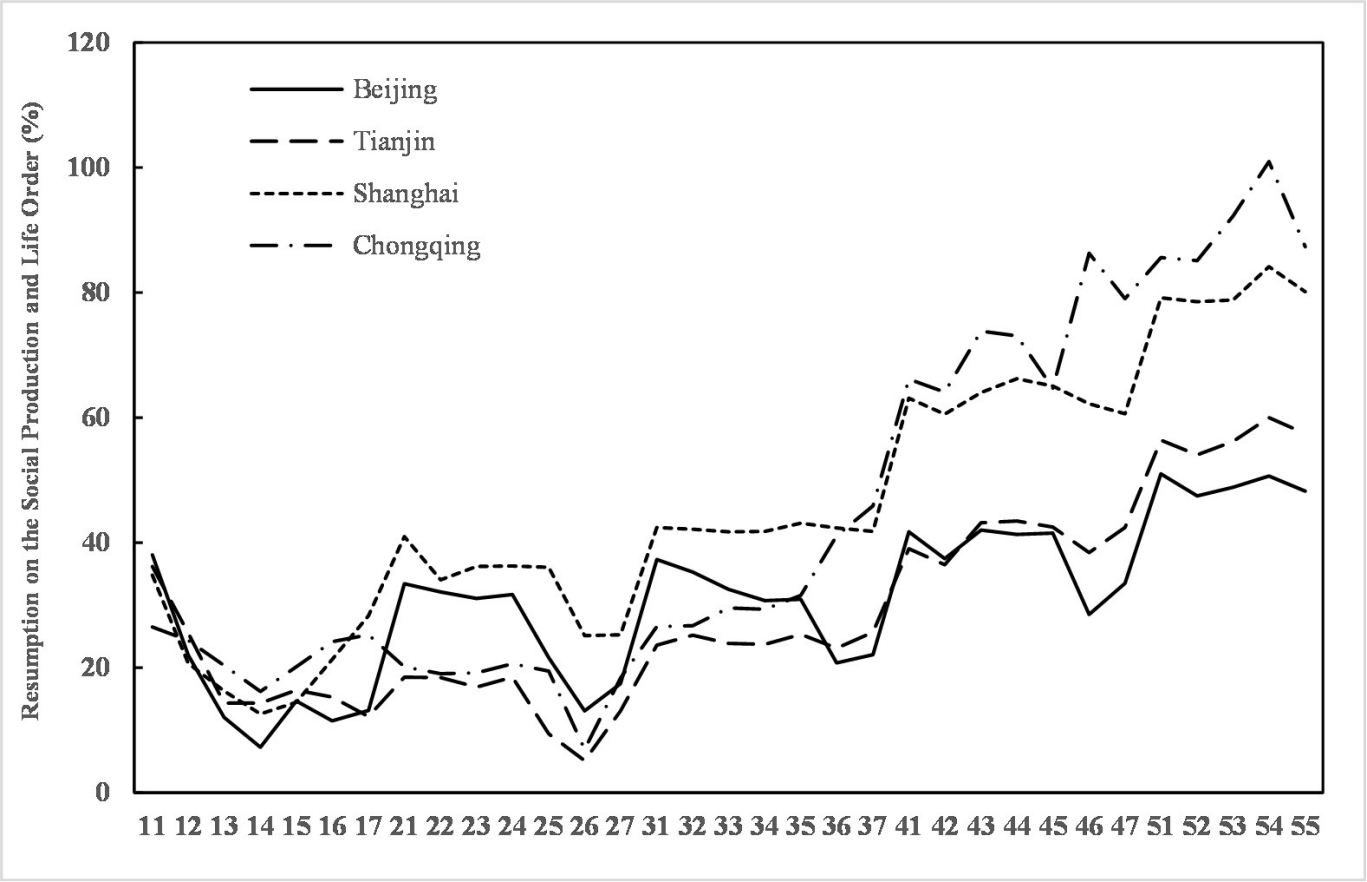
Changes in the resumption of social production and life in Beijing, Tianjin, Shanghai and Chongqing.

The population return scale and urban traffic can partly reflect the resumption of social production and life. After the Spring Festival holiday in 2020, the resumption of social production and life across the country shows a certain gap compared to 2019, and full resumption will be a gradual and step-by-step process. The level of resumption of social production and life in 13 cities and 25 provinces and municipalities exceeds 60%, and among the four municipalities, Beijing and Tianjin have lower resumption levels, 34.95% and 43.80%, respectively. The resumption levels of Shanghai and Chongqing are approximately 60%, reaching 61.63% and 63.40%, respectively. By the fifth week, resumption increases to its highest levels in Beijing, Tianjin, Shanghai, and Chongqing, 51.01%, 60.04%, 84.19%, and 101%, respectively, and Chongqing has the best resumption of social production and living order (Fig 6). Among the other 29 provincial capital cities, the resumption levels in Xining and Jinan are the highest (82.14% and 71.51%, respectively), whereas the resumption levels in Urumqi and Wuhan are the lowest, only 0.11% and 0.61%, respectively. In addition, Harbin and Hong Kong have very low resumption levels, 10.12% and 23.07%, respectively.

**Fig 7:**
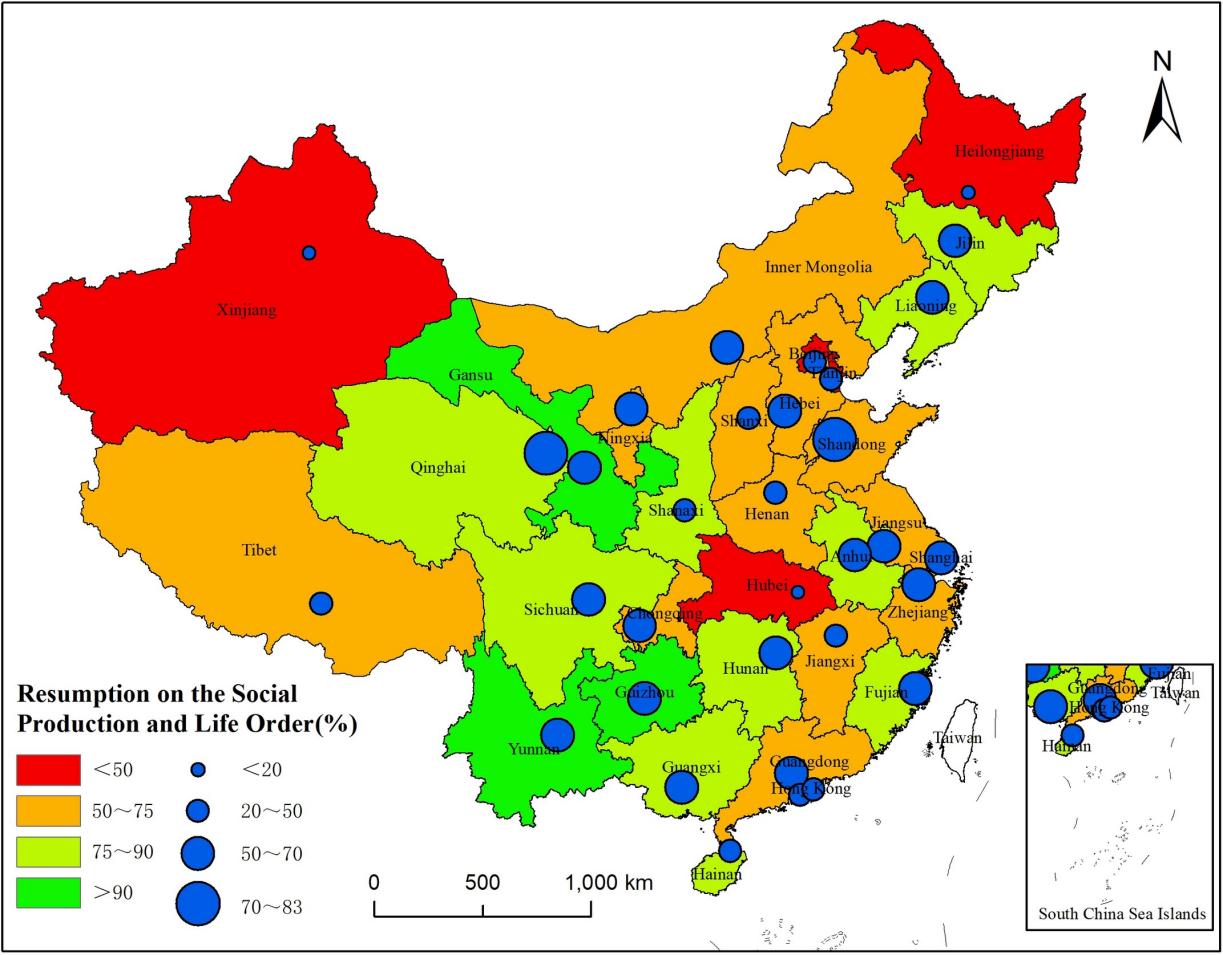
Distribution of the resumption of social production and life.

Fig 7 shows the overall resumption level of each province and city. Twelve out of 33 provinces and municipalities have resumed more than 80% of their social production and life. Among them, Guizhou and Yunnan are the two provinces with the highest resumption levels, 114.59% and 106.05%, respectively. Compared with 2019, the social production and life of the two provinces have nearly completely resumed. Notably, Gansu’s resumption level reaches 94.92%, a figure close to that of 2019. There are 6 places with resumption levels of less than 50%: Beijing, Tianjin, Xinjiang, Hubei, Hong Kong, and Macao. Among them, Xinjiang and Hubei have the lowest levels of resumption, 0.09% and 7.57%, respectively. In response to the resumption of social production and life in various places, especially in provinces and cities that are still in the process of resumption (having a resumption level below 50%), governments at all levels need to promote precise measures to fully resume social production and life.

### Analysis of driving factors

**Fig 8:**
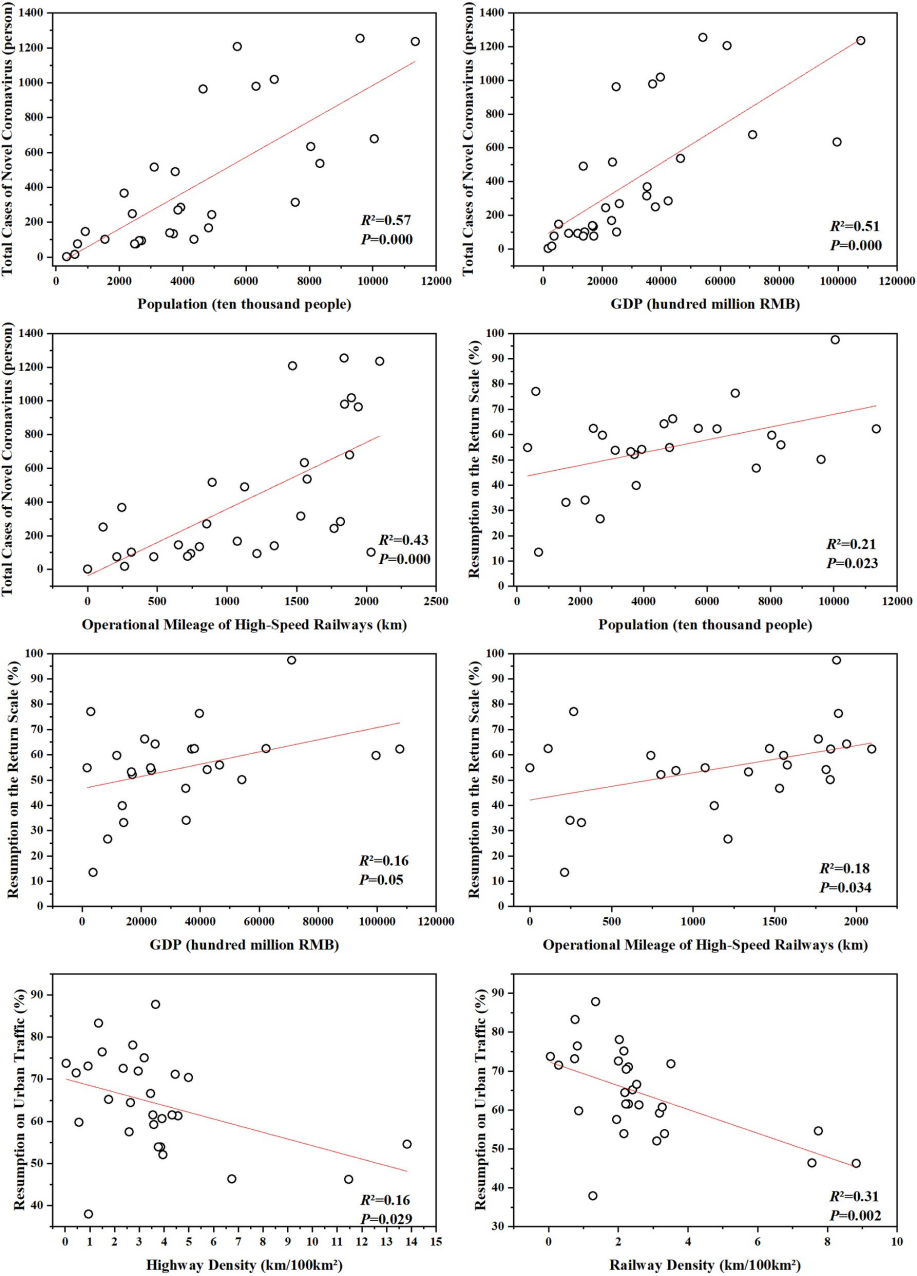
Correlation between the number of cases, resumption of return scale, resumption of urban traffic intensity and socioeconomic indicators.

The work resumption peak throughout the country appears in many provinces within one month of the end of the Spring Festival holiday. The results show that popular destinations are still first-tier cities, regional central cities and labor-intensive cities. For example, Beijing, Shanghai and Guangzhou are the cities most preferred by the returning population. Due to their sound economic foundation, large population and convenient transportation, these cities play a leading role in surrounding provinces. They produce a nonnegligible effect on the stable operation of the national economy and are also critical for the promotion of work resumption. In addition, the areas less affected by the epidemic are mainly provinces in southwest China (such as Tibet, Yunnan, Guizhou, and Sichuan), which have a lower level of urbanization and a relatively scattered population distribution. Furthermore, the transportation in these provinces is less developed, and the economic level and population mobility are lower than those in the developed regions in eastern China. To a certain extent, these factors prevented the spread of the epidemic. Therefore, we consider that socioeconomic factors may be important influencing factors for the distribution of confirmed cases, the population return scale and traffic intensity. We conducted a correlation analysis of the cumulative number of confirmed cases, the resumption of return scale, and the resumption of urban traffic intensity with GDP, population, high-speed rail mileage, and the railway and highway density in all provinces and cities (except Hubei Province) (see Fig 8). The number of cumulative confirmed cases has a very significant positive correlation with population, GDP, and high-speed rail mileage (P<0.01), indicating that regions with a denser population and a well-developed economy have a higher risk of cluster infection outbreaks. There is a weak positive correlation between the resumption of return scale and population, GDP, and high-speed rail mileage (P<0.05), indicating that population return in more densely populated and well-developed regions is in a positive trend. The government also has a great effect on epidemic prevention and socioeconomic development. It plays an active role in promoting the plan of work resumption and maintaining the stable operation of the national economy. There is a significant negative correlation between the resumption of urban traffic intensity and the density of highways and railways (P<0.05), indicating that the intensity of travel within provinces and cities with more developed intercity transportation is lower than that for the same period last year. The reason is the lockdown of cities, restrictions on travel and other relevant policies. In addition, residents are more aware of necessary methods, and they know the importance of staying home for epidemic prevention. These results show that the data used in this paper can adequately reflect the changes in population migration and traffic intensity during the epidemic. In the future, the government's epidemic prevention and work resumption methods should focus more on densely populated and economically developed provinces and cities, as a larger number of immigrants means a higher risk of bringing imported cases and cross infection incidents into a province.

## Conclusions and discussions

Population migration and urban traffic are two important components of the socioeconomic system. This paper uses big data from the Baidu Migration platform to analyze the return of population, urban traffic conditions, and the resumption of social production and life across China after the 2020 Spring Festival holiday. The main conclusions are as follows:

1. Provinces in eastern China have reported more cases than those in western China, and regions adjacent to Wuhan and Hubei Province have more confirmed COVID-19 cases than nonadjacent regions. Densely populated regions with well-developed economies and transportation are more likely to have cluster infection incidents.
2. The COVID-19 epidemic in 2020 has severely impacted the latter part of the Spring Festival travel rush (i.e., migrating people returning to their present places of residence), as shown by the significant reduction in the migration scale, along with the extended timespan and the uncertainty regarding the overall end of return. Among 33 provinces and municipalities, 70% (23 of them) have a population return scale that has resumed to a level less than 60%; for 39% (13 of them), the resumption level is less than 50%. Hubei, Hong Kong, Xinjiang, and Inner Mongolia have the lowest levels of population return, and their resumption levels are all below 5%. Hainan and Shandong have the highest levels of population return, 272.72% and 97.35%, respectively. Due to government regulations, the return of the population in densely populated and well-developed regions shows a positive trend.
3. The resumption of urban traffic under the epidemic is slow and varies greatly in different places. Among 33 provinces and municipalities, 22 have an urban traffic resumption level of more than 60%. Guizhou and Yunnan are the two provinces with the highest resumption levels, whereas. Xinjiang, Hubei, and Heilongjiang have the lowest resumption levels, only 29.37%, 35.76%, and 37.90%, respectively. Provinces and municipalities with well-developed intercity traffic have a lower level of resumption, mainly because of regulatory methods such as lockdowns and traffic restrictions. The increased public awareness of epidemic prevention and the decreased frequency of outdoor activities are also two positive factors in slowing the spread of the epidemic.
4. The resumption of social production and life across the country shows a certain gap compared to 2019, and full resumption will be a gradual and step-by-step process. Xining and Jinan have the highest levels of resumption, 82.14% and 71.51%, respectively, whereas Urumqi and Wuhan have the lowest levels of resumption, only 0.11% and 0.61%, respectively. Twelve out of 33 provinces and municipalities currently have resumption levels of more than 80%. Guizhou, Yunnan, and Gansu have the highest levels of resumption and have nearly resumed the work and life status of 2019, whereas Xinjiang and Hubei have the lowest resumption levels, only 0.09% and 7.57%, respectively. Thus, relevant government departments should focus more on densely populated and well-developed provinces and cities when applying epidemic prevention and work resumption methods.

Population migration and urban traffic are important components of the socioeconomic system. The foundation of the market economy lies in the full free flow of resources and elements, including people, materials and information. Thus, social and economic development is dependent on transportation and the relationship of demand. An increase in transportation demand is not only a sure result of social and economic development but also a fact that must be faced. Based on the scale of population return and the resumption of urban traffic, this paper analyzes the post-festival process of resuming social production and life under the COVID-19 epidemic, and it can provide a reference for local governments to make further decisions on the accurate promotion of production resumption at that time.
